# Supplementary material for: Association between car driving and successful ageing. A cross sectional study on the "S.AGES" cohort
Source: PLoS One. 2023 May 4;18(5):e0285313. doi: 10.1371/journal.pone.0285313 (PMC10159353; doi:10.1371/journal.pone.0285313)
Supplement: S1 Table — (DOC) [file pone.0285313.s002.doc]

**S2:** Successful ageing definition: difference between Young et al proposal and study definition

| **Variable to be measured** | **Young and al (1) measurement proposals** | **Definition in S.AGES** | **Success definition** |
| --- | --- | --- | --- |
| **Physiological component** | | | |
| **Comorbidity** | 18 chronic conditions  : angina, myocardial infarction, congestive heart  failure, peripheral arterial disease, hip fracture, osteoporosis,  osteoarthritis of hands, osteoarthritis of knees, osteoarthritis  of hips, rheumatoid arthritis, disc disease, spinal stenosis,  stroke, Parkinson’s disease, pulmonary disease, diabetes, hypertension,  and cancer. | history of stroke, heart disease, peripheric arterial disease, veinous thromboembolism, hypertension, Parkinson disease, thyroid dysfunction, type 2 diabetes, osteoarthrosis, osteoporosis, rheumatoid arthritis, chronic pain, cancer, liver dysfunction, peptic ulcer | A sum equal or inferior to 3 comorbidities (2). |
| **Functional impairment** | Nagi’s 7 physical performances variables (3) | Katz ADL (4) | Score of 6/6 |
|  |  | Lawton IADL (5) | Score of 4/4 |
|  |  | Professional care taker | Absence |
|  |  | Falls in the last 12 months | Absence |
| **Psychological component** | | | |
| **Cognitive function** | MMSE | MMSE (6) | MMSE ≥ 27 |
| **Emotional vitality** | having a high sense of personal mastery, being happy, and having low anxiety | None |  |
| **Geriatric depression** | GDS | GDS (7) | GDS < 10 (7) |
| **Social component** | | | |
| **Satisfaction with social engagement** | Sum of 5 items (satisfaction with contribution to community, satisfaction with respect by others, satisfaction amount of variety in life, satisfaction with the help received from family or friends, satisfaction with  the help given to family and friends) | None |  |
| **Spirituality or religious commitment** | Spirituality Index of Well-Being (SIWB). | None |  |
| **Social isolation** |  | Living condition (alone or not) | Not living alone (8,9) |
| ***Notes: MMSE = Mini Mental status examiniation, ADL = Activities of daily living, IADL = Instrumental activities of daily living, GDS = Geriatric Depressive Scalve*** | | | |
