# Supplementary material for: Association between car driving and successful ageing. A cross sectional study on the "S.AGES" cohort
Source: PLoS One. 2023 May 4;18(5):e0285313. doi: 10.1371/journal.pone.0285313 (PMC10159353; doi:10.1371/journal.pone.0285313)
Supplement: S2 Table — (DOC) [file pone.0285313.s003.doc]

**S3**: Missing values analysis: MMSE and or GDS missing subjects vs non MMSE nor GDS values

|  | **Total**  **N = 3434** | **Not missing values on MMSE nor GDS**  **2098(61.5)** | **Missing values on MMSE and/or MMSE 1311(38.5)** | **P value** |
| --- | --- | --- | --- | --- |
|  |  |
| **Socio-demographic parameters** |  |  |  |  |
| Observatory  Chronic pain  Atrial fibrillation  Type 2 diabetes | 1379(40.2)  1072(31.2)  983(28.6) | 861(40.8)  637(30.2)  611(29) | 518(39.1)  435(32.8)  372(28.1) | 0.268 |
| Age, mean(SD) | 77.6(6.2) | 77.2(6.2) | 78.3(6.2) | *< .001* |
| Apparent age |  |  |  |  |
| Less than chronological age | 779(22.7) | 515(24.4) | 264(19.9) | *< .001* |
| Equal to chronological age | 2304(67.1) | 403(66.5) | 901(68) |
| Greater than chronological age | 344(10) | 188(8.9) | 156(11.8) |
| Missing values | 7(0.2) | 3(0.1) | 4(0.3) |  |
| Sex, Females | 1945(56.6) | 1177(55.8) | 768(58) | *0.215* |
| ADL ^a^, mean(SD) | 5.8(0.6) | 5.9(0.4) | 5.7(0.7) | *< .001* |
| Missing values | 17(0.5) | 7(0.3) | 10(0.8) |  |
| IADL ^b^, mean(SD) | 3.6(0.9) | 3.7(0.7) | 3.4(1) | *< .001* |
| Missing values | 23(0.7) | 12(0.6) | 11(0.8) |  |
| Education level |  |  |  |  |
| Primary school | 1493(43.5) | 874(41.4) | 619(46.7) | 0.005 |
| Secondary school | 1226(35.7) | 775(36.7) | 451(34) |
| High school | 380(11.1) | 238(11.3) | 142(10.7) |
| University | 282(8.2) | 193(9.2) | 89(6.7) |
| Missing values | 53(1.5) | 29(1.4) | 24(1.8) |  |
| Professional status |  |  |  | *< .001* |
| Currently working or retired | 2780(81) | 1757(83.3) | 1023(77.2) |
| Missing values | 30(0.9) | 17(0.8) | 13(1) |  |
| Alcohol consumption | 877(25.5) | 555(26.3) | 322(24.3) | *0.206* |
| Missing values | 37(1.1) | 20(0.9) | 17(1.3) |
| Tobacco consumption |  |  |  |  |
| Never | 2544(74.1) | 1532(72.6) | 1012(76.4) | 0.01 |
| Former | 759(22.1) | 502(23.8) | 257(19.4) |
| Current | 110(3.2) | 64(3) | 46(3.5) |
| Missing values | 21(0.6) | 11(0.5) | 10(0.8) |
| Living area |  |  |  |  |
| Rural | 837(24.4) | 503(23.9) | 334(25.2) | *0.114* |
| Semi-rural | 823(24) | 531(25.2) | 292(22) |
| Urban | 1771(51.6) | 1075(51) | 696(52.5) |
| Missing values | 3(0.1) | 0(0) | 3(0.2) |  |
| Residency |  |  |  |  |
| Alone at home | 1216(35.4) | 720(34.1) | 308(25.1) | *0 .052* |
| Not alone at home | 2156(62.8) | 1358(64.4) | 798(60.2) |  |
| Living facility | 57(1.7) | 31(1.5) | 26(2) |  |
| Missing values | 5(0.1) | 0(0) | 5(0.4) |  |
| Professional caregiver | 744(21.7) | 413(19.6) | 331(25) | *< .001* |
| Missing values | 92(2.7) | 58(2.8) | 34(2.6) |
| Polypharmacy ^c^ | 2033(59.2) | 1292(61.3) | 741(55.9) | *0.002* |
| Comorbidities sum, mean(SD) | 3.6(1.6) | 3.6(1.5) | 3.4(1.6) | 0.415 |
| Missing values | 80(2.3) | 42(2) | 38(2.9) |  |
| Alzheimer disease | 653(19) | 399(18.9) | 254(19.2) | 0.804 |
| Missing values | 16(0.5) | 6(0.3) | 10(0.8) |  |
| Osteoarthritis | 1794(52.2) | 1127(53.4) | 667(50.3) | 0.092 |
| Missing values | 7(0.2) | 2(0.1) | 5(0.4) |  |
| Cancer | 449(13.1) | 269(12.8) | 180(13.6) | 0.469 |
| Missing values | 9(0.3) | 4(0.2) | 5(0.4) |  |
| Chronic pain | 2145(62.5) | 1361(64.5) | 784(59.2) | 0.002 |
| Missing values | 5(0.1) | 1(0) | 4(0.3) |  |
| Fall in the past year | 383(11.2) | 211(10) | 172(13) | 0.006 |
| Missing values | 21(0.6) | 7(0.3) | 14(1.1) |  |
| Peptic ulcer history | 112(3.3) | 66(3.1) | 46(3.5) | 0.514 |
| Missing values | 13(0.4) | 6(0.3) | 7(0.5) |  |
| Heart condition | 1568(45.7) | 936(44.4) | 632(47.7) | 0.043 |
| Missing values | 30(0.9) | 15(0.7) | 15(1.1) |  |
| Hypertension | 2397(69.8) | 1486(70.5) | 911(68.8) | 0.383 |
| Missing values | 14(0.4) | 5(0.2) | 9(0.7) |  |
| Liver condition | 63(1.8) | 36(1.7) | 27(2) | 0.475 |
| Missing values | 12(0.3) | 5(0.2) | 7(0.5) |  |
| Osteoporosis | 491(14.3) | 303(14.4) | 188(14.2 | 0.939 |
| Missing values | 10(0.5) | 5(0.6) | 5(0.4) |  |
| Type II diabetes | 1395(40.6) | 859(40.7) | 536(40.5) | 0.953 |
| Missing values | 9(0.3) | 2(0.1) | 7(0.5) |  |
| Thyroid dysfunction | 428(12.5) | 268(12.7) | 160(12.1) | 0.602 |
| Missing values | 19(0.9) | 7(0.8) | 12(1) |  |
| Parkinson disease | 45(1.3) | 28(1.3) | 17(1.3) | 0.922 |
| Missing values | 12(0.3) | 4(0.2) | 8(0.6) |  |
| Pulmonary condition | 393(11.4) | 256(12.1) | 137(10.3) | 0.115 |
| Missing values | 4(0.2) | 1(0.1) | 3(0.2) |  |
| Rheumatoid arthritis | 169(4.9) | 106(5) | 63(4.8) | 0.746 |
| Missing values | 15(0.4) | 5(0.2) | 10(0.8) |  |
| Stroke | 93(2.7) | 46(2.2) | 47(3.5) | 0.015 |
| Missing values | 16(0.5) | 6(0.3) | 10(0.8) |  |
| Thromboembolic history | 248(7.2) | 143(6.8) | 105(7.9) | 0.197 |
| Missing values | 37(1.1) | 20(0.9) | 17(1.3) |  |
| **Successful ageing components** |  |  |  |  |
| Physiological component | 1106 (32.2) | 702 (33.3) | 404 (30.5) | < 0.052 |
| Missing values | 1596 (46.5) | 910 (43.1) | 62 (5.1) |  |
| Social ccomponent | 2213 (64.4) | 1389 (65.9) | 824 (62.2) | < .041 |
| | ***Note. Data are number (%) unless otherwise indicated. In case of no missing value, the line empty was kept empty.***  ***^a^ADL= Activities of daily living, ^b^IADL = Instrumental activities of daily, ^c^Polypharmacy*  ≥ 5 treatments** | | --- | | | | | |
