# Supplementary material for: Association between car driving and successful ageing. A cross sectional study on the "S.AGES" cohort
Source: PLoS One. 2023 May 4;18(5):e0285313. doi: 10.1371/journal.pone.0285313 (PMC10159353; doi:10.1371/journal.pone.0285313)
Supplement: S3 Table — (DOC) [file pone.0285313.s004.doc]

**S4: Table of Heart diseases, Pulmonary and Liver condition details among drivers and non- drivers**

|  | **Total** | **Non-drivers** | **Drivers** |  |
| --- | --- | --- | --- | --- |
|  | **N = 2098** | **872 (41.6%)** | **1226 (58.4%)** | p |
| Sleep Apnea | 84 (4) | 22 (2.5) | 62 (5.1) | 0.004 |
| Missing values | 5 (0.2) | 2 (0.2) | 3 (0.2) |  |
| COPD/Fibrosis*^a^* | 190 (9.1) | 78 (8.9) | 112 (9.1) | 0.869 |
| Missing values | 5 (0.2) | 1 (0.1) | 4 (0.3) |  |
| Other arrhythmias*^b^* | 80 (3.8) | 36 (4.1) | 44 (3.6) | 0.532 |
| Missing values | 15 (0.7) | 5 (0.6) | 10 (0.8) |  |
| Implantable cardioverter-defibrillator | 5 (0.2) | 1 (0.1) | 4 (0.3) | 0.408 |
| Missing values | 18 (0.9) | 4 (0.5) | 14 (1.1) |  |
| AF*^c^* | 763 (36.4) | 284 (32.6) | 479 (39.1) | 0.002 |
| Missing values | 3 (0.1) | 1 (0.1) | 2 (0.2) |  |
| Heart failure | 243 (11.6) | 118 (13.5) | 125 (10.2) | 0.021 |
| Missing values | 17 (0.8) | 4 (0.5) | 13 (1.1) |  |
| Pacemaker | 95 (4.5) | 36 (4.1) | 59 (4.8) | 0.44 |
| Missing values | 17 (0.8) | 4 (0.5) | 13 (1.1) |  |
| Valvulopathy | 54 (2.6) | 15 (1.7) | 39 (3.2) | 0.035 |
| Missing values | 18 (0.9) | 4 (0.5) | 14 (1.1) |  |
| Cirrhosis, | 11 (0.5) | 5 (0.6) | 6 (0.5) | 0.77 |
| Missing values | 6 (0.3) | 2 (0.2) | 4 (0.3) |  |
| Liver cytolysis | 27 (1.3) | 9 (1) | 18 (1.5) | 0.382 |
| Missing values | 5 (0.2) | 2 (0.2) | 3 (0.2) |  |
| *Note. Data are number (%) unless otherwise indicated. In case of no missing value, the line empty was kept empty.*  *^a^COPD= Chronic obstructive pulmory disease, ^b^Other arrythmias = Ventricular arythmia and/or torsade de pointe, ^c^ FA =*  *Atrial fibrillation* | | | | |
