# Supplementary material for: Association between car driving and successful ageing. A cross sectional study on the "S.AGES" cohort
Source: PLoS One. 2023 May 4;18(5):e0285313. doi: 10.1371/journal.pone.0285313 (PMC10159353; doi:10.1371/journal.pone.0285313)
Supplement: S1 File — (DOCX) [file pone.0285313.s005.docx]

S 5 : Confusion matrix of driving status and successful ageing:

Prediction of driving status according to successful ageing

|  |  | Prediction of driving status | |
| --- | --- | --- | --- |
|  |  | Yes | No |
| Driving status | Yes | 762 (38.9%) | 59 (3%) |
|  | No | 849 (43.4%) | 287 (14.7%) |

Sensitity = 0.75
Specifity = 0.83
Area Under the Curve = 0. 86
