# Supplementary material for: Association between car driving and successful ageing. A cross sectional study on the "S.AGES" cohort
Source: PLoS One. 2023 May 4;18(5):e0285313. doi: 10.1371/journal.pone.0285313 (PMC10159353; doi:10.1371/journal.pone.0285313)
Supplement: Attachment — Submitted filename: Reviewer_final.docx [file pone.0285313.s006.docx]

Reviewer #1: Dear authors, thank you for your efforts and job.
In my opinion, the manuscript is a little bit too weak for the journal of PlosOne.
The abstract of this manuscript is not very informative, I mean e.g. SA. There is no clear criteria's what is SA, just a reference to Young et al definition. It is quite difficult when one needs to look for other sources, that to understand what is SA. The part of the methodology is described extensively, I recommend using a more structured style.
In the introduction and in general, there is too little justification as to how SA relates to driving.

**Response:**

We thank you for your relevant comments. The abstract has been rewritten and restructured in order to make it more informative regarding successful ageing and Young et al definition. The following sentences were added:

- Line 25: “SA is comprised of 3 dimensions: an active engagement with life, a low probability of disease and disease-related disability, and a high cognitive and physical functional capacity. Driving seems to be linked to SA as it is a mean to preserve social interactions and it requires preserved functional and cognitive status.”

- Line 31: SA was defined by the success of three dimensions: physiological comprised of co-morbidity and autonomy scores, psychological comprised of cognitive status and emotional state, and a social dimension (social isolation).

The general content and the introduction have been rewritten for the relationship between SA and driving to be clearer. The following sentences were changed:

- Line 79: Nonetheless, driving also represents a major factor for our elders’ social interaction as shown on a prospective cohort of 4,359 community-dwelling older adults (mean age 78.72 (7.32) y/o): frequent drivers are most likely to visit friends and family (odds ratio (OR) 1.75; p < .01), go out for enjoyment (1.75; p <.001), attend to religious services (1.77; p <.01) compared with subjects who ceased driving (6). However, driving represents a complex activity as it implies multiple simultaneous tasks with different temporal and cognitive requirements (7). Finally, it requires one to be physically fit as it requires good sight, coordination, muscle power and control (8). ‬‬‬‬‬‬

- Line 87: These three aspects (sociological, cognitive and functional) are parts of successful aging (SA) as theorized by Rowe and Kahn which is defined as: an active engagement with life, a low probability of disease and disease-related disability, and a high cognitive and physical functional capacity (9). In fact, on a large cohort of 2475 subjects, based on a 0-10 SA score (the higher the better), it has been shown that drivers had higher SA score (OR 0.65 [0.54–0.77]) (10).

- Line 117: However, it has been pointed out on a systematic review (11) that association between driving and successful ageing is yet poorly understood and that there is an under-representation of mean of transports in studies on SA.

Reviewer #2: Comments to Authors:

Overview:
The present study aimed to assess driving determinants and association between driving status and successful aging among participants aged 65 or over. The design of a study was a cross-sectional. The topic analysed in the paper is relevant and important, however, there is some drawbacks that need to be addressed.

− Introduction is very general and lacks specific background on the problem/topic analysed in the study. The justification for the study could be stronger.
− Discussion lacks practical implications of the study findings, which is crucial for the paper. Although some statements and sentences in the discussion refer to relationships which cannot be based on a cross-sectional analysis.
− Gender, education level and living area (rural/urban) specific discussion would be useful based on the results.
− Authors could offer practical recommendations to improve successful aging.

Minor comments:
− Page 5, line 113: MMSE abbreviation is not explained.
− Page 6, line 137: “… a stepwise was performed.” Maybe stepwise regression?
− Page 6, line 139: ”…confidence interval OR [CI].” I guess the OR is not needed here.
− Page 7, line 159: “75. 6” there is unnecessary space.
− Page 11, line 186: “This work found that driving was associated with driving status” ?

**Reponse**: We thank you for these relevant comments. Introduction and background have been reviewed and rewritten in order to entrench implications:

- Line 79: Nonetheless, driving also represents a major factor for our elders’ social interaction as shown on a prospective cohort of 4,359 community-dwelling older adults (mean age 78.72 (7.32) y/o): frequent drivers are most likely to visit friends and family (odds ratio (OR) 1.75; p < .01), go out for enjoyment (1.75; p <.001), attend to religious services (1.77; p <.01) compared with subjects who ceased driving (6). However, driving represents a complex activity as it implies multiple simultaneous tasks with different temporal and cognitive requirements (7). Finally, it requires one to be physically fit as it requires good sight, coordination, muscle power and control (8). These three aspects (sociological, cognitive and functional) are parts of successful aging (SA).

The discussion has also been reviewed in order to offer practical application of the study findings and successful ageing improvement:

- Line 442: This calls for ways to decrease the rates of drivers suffering from conditions with high risk of accident such as a regular screening of driving skills (psychomotor and cognitive abilities) with the use of simulator or virtual reality.
- Line 446: Nonetheless, regarding these results, in order to maintain sociological interactions and to reach SA, we advocate for our elder’s mobility. This may be achieved either by improving preventive medicine and comprehensive geriatric assessment in order to improve physiological and psychological components. In case of irreversible pathological state, instead of developing of public transport, development of special transport services and communication of their existence (half of respondents of a large Norwegian survey knew about these services (28)), or maybe the use of, soon available, driverless cars (42) might be solutions for the impaired 65+.

We also thank the reviewers for the minor comments which where acknowledged:

- Line 183: Cognitive function was assessed by Mini Mental State Evaluation (MMSE)
- Line 225: Then, a stepwise logistic regression […]
- Line 223: Results are presented with odds ratios and 95% confidence interval (CI).
