# Supplementary material for: Association between car driving and successful ageing. A cross sectional study on the "S.AGES" cohort
Source: PLoS One. 2023 May 4;18(5):e0285313. doi: 10.1371/journal.pone.0285313 (PMC10159353; doi:10.1371/journal.pone.0285313)
Supplement: Attachment — Submitted filename: Review PONE D-22-26799_UBC.docx [file pone.0285313.s007.docx]

PONE-D-22-26799

“Association between car driving and successful ageing. A cross sectional study on the "S.AGES" cohort” by E. Baudouin and colleagues

The manuscript to be reviewed investigates whether driving status of aged individuals can be considered as a proxy of successful ageing (SA), comprising physical, psychological and social factors. It included non-drivers or drivers aged 65 and above. The study-cohort is part of the larger cross-sectional study S.AGES (Sujets AGÉS—Aged Subjects), an observational prospective cohort study looking at General Practicioner´s (GP) management of patients suffering from three chronical physical conditions, namely arterial fibrillation, chronic pain and diabetes mellitus type 2. Overall, it is scholarly written and informative. The topic is worth investigating.

Nevertheless, I have a couple of comments the authors may consider to specify some aspects to improve their work.

The model of successful ageing (SA) by Rowe and Khan referred to in the study manuscript is important but still one of multiple definitions of successful ageing that do exist (see review in Estebsari F. et al Current Aging Science, 2020, 13, 4-10), and has been refined and extended meanwhile by some authors (e.g. 2002 by Crowther and others). Rowe and Khan have published their model in 1997, not 1987 as mentioned in the text (Rowe JW, Kahn RL. Successful aging. Gerontologist. 1997 Aug;37(4):433-40. doi: 10.1093/geront/37.4.433. PMID: 9279031.) Relevant citations missing in the manuscript. This should be added and outlined.

E.g. Almeida et al. (2006) defined successful aging according to Mini-Mental State Examination (MMSE) as scoring 24 and above, and to Geriatric Depression Scale (GDS) as scoring 5 and below. Hence, the authors of the study under revision rightly used a more restrictive definition with regard to the cognitive status (MMSE <27). Even more so, as the MMSE alone is absolutely insufficient to evaluate a patient’s driving competence.

In contrast, subjects with a GDS score below 10 are regarded as psychologically healthy, whereas Yesavage et al. (1983), author of the GDS, defined less than 5 points as “no depression” and 5-10 points as “mild to moderate” depression. It is well accepted that somatic comorbidity is a risk factor for depression. Both aspects may affect the results of the study. A more restricted threshold for depressive symptoms is preferable, or the authors may have to explain why they decided otherwise.

The study only included patients > 65 yrs. with somatic disorders (chronic pain, diabetes mellitus and atrial fibrillation) not healthy community-dwelling subjects, a selection bias which should be referred to, even if most older people may suffer from one or the other chronic somatic disorder.

It does not become clear how GPs who followed up on the study-participants measured the physiological age being lower, equal or higher to the patients´ chronological age. Did they use objective measures or subjective impressions prone to low interrater reliability. This should be outlined in the text.

Looking at the three sub-cohort´s results and inclusion characteristics it is worthwhile mentioning that atrial fibrillation and type II diabetes do not seem to impact on the driving status (more drivers with this condition than non-drivers), whereas chronic pain is equally distributed in both groups. Is chronic pain an independent risk factor for reduced successful aging and giving up driving?

The three subcomponents of the successful aging model by the Rowe and Khan refined by Young et al 2009 are weighed unequally in this study, as it is dominated by aspects on the physiological component (a combined factor out of 19 items in the comorbidity dimension plus the autonomy dimension, such as ADL and IADL). Whereas very cursory aspects of cognition and mood (MMSE and GDS<10) for the psychological component and even more superficial on the sociological component, (only represented by the question whether the participant lived alone or not, which may have many different reasons without necessarily meaning that a person is socially isolated or not), have been applied.

Unfortunately, a conclusive measure of quality of life such as HRQOL relevant for successful ageing and driving status is missing (Hajek A 2021).

To sum up, the manuscript “Association between car driving and successful ageing. A cross sectional study on the "S.AGES" cohort” by E. Baudouin and colleagues may pose a relevant contribution to the scientific field of successful ageing. It is in line with the current literature supporting the view that driving is a relevant aspect to wellbeing until older age (in industrialised countries). The shortcomings are most likely due to the fact that the study was not primarily designed to answer the question of successful ageing but to look at GPs management of chronic conditions namely diabetes mellitus, chronic pain and atrial fibrillation. A fact that is outlined by the authors in the text and will be evaluated by the editors. If this is considered suitable, after minor revision taking into account the remarks made above a publication in PLOS One can be recommended.

Kind regards and

bonne chance!
