# Supplementary material for: Association between car driving and successful ageing. A cross sectional study on the "S.AGES" cohort
Source: PLoS One. 2023 May 4;18(5):e0285313. doi: 10.1371/journal.pone.0285313 (PMC10159353; doi:10.1371/journal.pone.0285313)
Supplement: Attachment — Submitted filename: Response to Reviewers.docx [file pone.0285313.s008.docx]

**Reviewer #1**

Thank you for your relevant comments. We addressed all the questions that you raised and highlighted it in the text in blue.

Please find enclosed our answers
Major concerns:
**1. The patients suffer from one of 3 diseases, which should be mentioned already here and also in the title, which suggests that the study deals with healthy elderly.**

We modified the methods part of the abstract in order to precise that included patients were suffering from suffering from chronic pain, type-2 diabetes mellitus or atrial fibrillation:

“This cross-sectional study is an ancillary study from the S.AGES (Sujets AGÉS—Aged Subjects) study, an observational prospective cohort study which included patients suffering from chronic pain, type-2 diabetes mellitus or atrial fibrillation from 2009 to 2014”.

**2. Driving behavior and success are not included, just driving vs. nondriving.**

Details:
l.30: unclear, what determinants? No driving determinants are measures, only driving or not driving. The simple finding is that drivers are more often successful agers than nondrivers.

We modified l.30 in order to precise that the study is about driving status: “This study aims to investigate if driving status can be considered as a proxy of SA, by describing determinants associated with driving status in the 65+.”

L42: better: it reflects.

L43 has been corrected as per your suggestion:” Driving may be considered as a proxy to SA: it reflects elders’[…]”

l.74: determinants? better:... driving status.

L82 has been corrected: “Therefore, the aim of this study was to investigate if driving status can be considered as a proxy of SA, by describing driving status determinants in the 65+.”

l.106: AF was the condition of one of the 3 groups

Indeed, AF was a selection condition for 636 (30.3%) of the patients. However, when looking at AF prevalence in this cohort, we find that this affliction is still present in 763 patients (36.4%). We added the “S4 Table” which details heart condition, liver diseases and pulmonary condition in the cohort.

l.123: the MMSE is a very superficial and unspecific measure for the various cognitive functions. It is not suitable to measure cognition in healthy elderly and is more appropriate to check for MCI or dementia.

We thank you for your remark. MMSE is indeed superficial and unspecific measure for the various cognitive functions. However, it is used in routine as a screening tool and as a mean to assess cognitive function in successful ageing literature (Cevenini E, Cotichini R, Stazi MA, Toccaceli V, Scurti M, Mari V, et al. How to classify the oldest old according to their health status: A study on 1160 subjects belonging to 552 90+ Italian sib-ships characterized by familial longevity recruited within the GEHA EU Project. Mech Ageing Dev. nov 2013;134(11‑12):560‑9).

l.229: some text is missing after “any”.

Thank you. This has been corrected “[…] only few people are free from any affliction”
l.256: here more possibilities should be mentioned to improve driving capabilities of older drivers, such as physical and cognitive training, as well as driving training.

**Reviewer #2**:

Thank you for your relevant comments. We addressed all the questions that you raised and highlighted it in the text in yellow.

In the abstract, percentage should be given with the associated ratio since not of the data presented match in terms of calculations and percentage shown.

We thank you for this remark. The results in the abstract have been changed as such: “351/2,092 (16.7%) were classified as successful agers: 292/1,266 (23.8%) in the drivers group vs 59/872 (6.8%) in the non-drivers; p <.001”

Line 51: participation of older persons in traffic should be presented as a prevalence and not older drivers generating traffic on the road.
Line 53: Results from France would be better than the USA to highlight the relative risk of older drivers even though these results might dated.
Line 55: there is an error with the percentage sign (%)

We have modified to rational in order to provide French statistics regarding road safety and old adults involvments in car crash. L57: “However, the 65+ years old (y/o) represents a risk regarding road safety with motor vehicle crash death: in 2021 13% (383 car drivers out of 2944 road fatalities) when considering the 65+ in France according to the French National observatory of road safety”

Line 88: how is physiological age defined (and reference) ? Since it is one of the main metric, it has to be better defined herein.

We have added precision and reference on physiological age: L98 GPs were asked to classify each patient according to his/her apparent age into one of three categories by answering the question: "In your opinion, is this patient's apparent age younger than, the same as, or older than his or her chronological age?(12).

In Table 1, with the age of the sample, how can we be sure that the high percentage of women who are not driving (84.5%) is actually associated with health or social status while they might have never had a driver license from the beginning? It is not uncommon at the age to see older women who never drove and therefore make it difficult to account for driving as a successful indicator of SA if one might have never done it before.
This is raised on line 215. For line 216, this sentence should be supported by your results and not the one from the reference 29.

Indeed, this association can only be assessed in people who had the opportunity to drive in the first place. However, this may impact the correlation between SA and driving but not the results of SA itself as discussed in line 248. We also added this section L246: This result is found in this cohort regarding comorbidities and driving status differences between men and women (S5 Table) but is also consistent with literature regarding SA (31,32), where men are more frequently classified as successful agers irrespective of the SA definition used

Why is professional status merged for “Currently working or retired”?

Only 6 subjects were currently working in the cohort. We assume the impact of having a work history of working to be closer to the one currently working than the retired subjects. This precision was added in the result section L 187: Finally, because only 6 subjects had responded “Currently working” regarding their professional activity, they were merged with the retired subjects as opposed to the “never had a professional activity” group.

Line 205 : the vs needs non-driving group after for clarity.

Thank you, this has been changed accordingly.

Line 242 (presented as 442 in the response to reviewers) lacks scientific support in this domain in regards of evaluation and screening for at risk drivers and would merit a more detailed discussion (See Brenda Vrjklan’s work or Michel Bedard for more details).

We thank you for this advice. We have completed the discussion (L283) with more scientific background: “Moreover, development of tools to screen for drivers with cognitive impairment is needed such as Screen for the Identification of Cognitively Impaired Medically At-Risk Drivers or Trail Making Test have shown high rate of misclassification on pass/fail on a road test in drivers (42,43). It is however interesting to note that according to a Canadian study of 108 older adults (mean age 80.6 +/- 4.9 y/o) on self-awareness of driving performance, 53% over-estimate themselves (vs 19% of underestimation).”

Line 211 raises an important issue, if driving seems closely link with urban vs rural area, why would not cessation of driving be seen as SA if one is voluntarily transitioning from car ownership to public and active transportation? You refer to it on line 269. Your argument should be consistent.

In order for the argumentation to be clearer, the sentence (L297) has been changed to […]instead of developing common public transport[…]

Line 246 (not Line 446): Public transport and its associated services cannot be placed in contradiction with autonomous vehicles. The latest have been sold has the new panacea for older drivers for the last 10 years despite showing real improvement for them. Moreover, the driverless car also neglects the last 30 feet (10 mètres) that one will have to cover from curbside to house or services. Therefore, they still have to be thought as a form of public transportation and should be presented as so.

It was not intended to place autonomous vehicles in contradiction with autonomous vehicles. Moreover, we referred for personal autonomous vehicles which are still under development and could be closer to personal vehicle than a public transport service. The sentence L301 has been rephrased […]“or maybe the use of driverless cars in association with public transports (46) might be solutions for the impaired 65+.”

Regarding identification of at-risk drivers and maintenance of one’s license, a clearer portrait of the France license continuum should be made because it is hard to understand why you have 23% of the drivers who present MMSE under 27 (see Table 1), full or average score obtained should be given to better present the sample. From line 238 to 245, solutions should be identified to alleviate this limitation from the results. Moreover, the opening of the paragraph is hypothetical since and should be used with caution since there is no data from the study or from other French study to support it.

Thank you for this remark. A clearer portrait of France license policy has been added line and updated (L. 272) “This result might be found in this cohort mainly because in France there is no expiration date of the driving license and medical confidentiality must be maintained. Since fall 2022, when one suffers from a specific group of diseases (including cognitive impairment), it is required to get a medical approval by a certified physician. » However, this particular medical appointment can only be made by the patient himself after receiving information on the risk and obligation regarding his condition; medical conditions cannot be disclosed by any health care professional. Moreover, we added MMSE mean of the two groups in the result section L 191: “[…] and a mean MMSE of 27.9 (2.4) vs 26.7 (2.9) in the non-drivers group; p < .001.”

Line 266: social interaction (not sociological), change elsewhere

Thank you, this has been corrected

References should be checked to ensure that they are all presented in English (ex. Février, éditeurs, cite, etc.).

Thank you, references have been updated accordingly.

As a limitation, it should be noted that the sample is built upon a subsample of a study which aimed at “describe therapeutic management of outpatients”. Therefore, there is higher chance that the sample present medical diagnostic that might not be compatible with driving.

Thank you, this has been acknowledged in the limit section (L 301). “First, this study concerned 65+ suffering from a chronic disease (AF, chronic pain or type 2 diabetes) with regular follow up with a physician. However, this study is still relevant as these three pathologies represent a high prevalence in Europe and worldwide, especially in the 65+ population (45–47)”

Finally, would there be an opportunity to use some other factors as proxy to identify faster SA? As an example, only using the IADL use of mean of transport ?

Indeed, this could be another way to assess for SA. Nonetheless, it was found that driving also correlates well with health-related quality of life (Hajek et al, 2021) which was added in the discussion section (L276). Specific work on these two measures might be of interest in order to differentiate their usage.

Overall, as a clinical perspective, a confusion matrix should be used in order to identify who was or was not classified correctly as drivers and SA (or not).

We have added a confusion matrix in “Confusion matrix and parameters in term of sensitivity, specificity and area under the curve can be found in supplementary S 5 Confusion matrix of SA and Driving status.”

**Reviewer #3**: PONE-D-22-26799

Thank you for your relevant comments. We addressed all the questions that you raised and highlighted it in the text in purple.

The model of successful ageing (SA) by Rowe and Khan referred to in the study manuscript is important but still one of multiple definitions of successful ageing that do exist (see review in Estebsari F. et al Current Aging Science, 2020, 13, 4-10), and has been refined and extended meanwhile by some authors (e.g. 2002 by Crowther and others). Rowe and Khan have published their model in 1997, not 1987 as mentioned in the text (Rowe JW, Kahn RL. Successful aging. Gerontologist. 1997 Aug;37(4):433-40. doi: 10.1093/geront/37.4.433. PMID: 9279031.) Relevant citations missing in the manuscript. This should be added and outlined.

We thank you for this remark. The citation has been corrected accordingly.

E.g. Almeida et al. (2006) defined successful aging according to Mini-Mental State Examination (MMSE) as scoring 24 and above, and to Geriatric Depression Scale (GDS) as scoring 5 and below. Hence, the authors of the study under revision rightly used a more restrictive definition with regard to the cognitive status (MMSE <27). Even more so, as the MMSE alone is absolutely insufficient to evaluate a patient’s driving competence.
In contrast, subjects with a GDS score below 10 are regarded as psychologically healthy, whereas Yesavage et al. (1983), author of the GDS, defined less than 5 points as “no depression” and 5-10 points as “mild to moderate” depression. It is well accepted that somatic comorbidity is a risk factor for depression. Both aspects may affect the results of the study. A more restricted threshold for depressive symptoms is preferable, or the authors may have to explain why they decided otherwise.

Thank you for this important observation. Indeed, GDS authors defined that less than 5 points as “no depression” and 5-10 points as “mild to moderate” depression. However, this has been validated on a 55+ cohort. Blank et al on a cohort of primary care patients, mean age 76.8 ± 8.9 y/o showed that a cut-off < 10 had better parameters that a cut-off of 5. We found this cohort to be closer to ours than Yesavage et al which is why this cut-off was used. This has been added to the method section line 143 with appropriate citation.

The study only included patients > 65 yrs. with somatic disorders (chronic pain, diabetes mellitus and atrial fibrillation) not healthy community-dwelling subjects, a selection bias which should be referred to, even if most older people may suffer from one or the other chronic somatic disorder.

Thank you for this comment. This has been assessed in the limit section “First, this study concerned 65+ suffering from a chronic disease (AF, chronic pain or type 2 diabetes) with regular follow up with a physician. However, this study is still relevant as these three pathologies represent a high prevalence in Europe and worldwide, especially in the 65+ population (45–47)”

It does not become clear how GPs who followed up on the study-participants measured the physiological age being lower, equal or higher to the patients´ chronological age. Did they use objective measures or subjective impressions prone to low interrater reliability. This should be outlined in the text.

We have added precision and reference on physiological age: L116 GPs were asked to classify each patient according to his/her apparent age into one of three categories by answering the question: "In your opinion, is this patient's apparent age younger than, the same as, or older than his or her chronological age?(12).

Looking at the three sub-cohort´s results and inclusion characteristics it is worthwhile mentioning that atrial fibrillation and type II diabetes do not seem to impact on the driving status (more drivers with this condition than non-drivers), whereas chronic pain is equally distributed in both groups. Is chronic pain an independent risk factor for reduced successful aging and giving up driving?

Thank you for this observation. This seems to be found indeed on the sub-cohorts. However, this result is not found in multivariate analysis after adjustment on the same variables tried on the stepwise regression as the main model.

|  | ***Odds Ratios*** | ***CI*** | ***p*** |
| --- | --- | --- | --- |
| Atrial fibrillation (ref = No) | 0.87 | 0.66 - 1.15 | 0.3 |
| Type II diabetes (ref = No) | 0.89 | 0.69 - 1.16 | 0.4 |
| **Chronic pain (ref = No)** | 0.80 | 0.61 - 1.06 | 0.13 |
| Age | 0.89 | 0.87 - 0.91 | <0.001 |
| Sex (ref = F) | 6.55 | 4.80 - 9.00 | <0.001 |
| Education level (ref = primary school) |  |  |  |
| Secondary school | 2.11 | 1.63 - 2.72 | <0.001 |
| High school | 2.97 | 2.00 - 4.42 | <0.001 |
| University | 2.77 | 1.78 - 4.36 | <0.001 |
| Professional status (ref = never employed) | 1.71 | 1.25 - 2.35 | <0.001 |
| Matrimonial status (ref = bachelor) |  |  |  |
| Living with a partner | 1.10 | 0.72 - 1.68 | 0.6 |
| Widower | 0.78 | 0.50 - 1.20 | 0.3 |
| Living area (ref = urban) |  |  |  |
| Rural | 2.95 | 2.20 - 3.98 | <0.001 |
| Semi-rural | 2.21 | 1.67 - 2.93 | <0.001 |
| Alcohol consumption (ref = no) | 1.68 | 1.24 - 2.29 | <0.001 |
| Polypharmacy (ref = No) | 0.85 | 0.67 - 1.08 | 0.2 |
| Physiological age (ref = equal to chronological age) |  |  |  |
| Less than chronological age | 1.35 | 1.03 - 1.77 | 0.029 |
| More than chronological age | 0.24 | 0.16 - 0.36 | <0.001 |
| Tobacco consumption (ref = never) |  |  |  |
| Former | 1.49 | 1.07 - 2.09 | 0.019 |
| Curent | 0.99 | 0.51 - 1.94 | >0.9 |
| *Notes : OR = Odds Ratio, ^a^polypharmacy ≥ 5 treatments* | | | |

The three subcomponents of the successful aging model by the Rowe and Khan refined by Young et al 2009 are weighed unequally in this study, as it is dominated by aspects on the physiological component (a combined factor out of 19 items in the comorbidity dimension plus the autonomy dimension, such as ADL and IADL). Whereas very cursory aspects of cognition and mood (MMSE and GDS<10) for the psychological component and even more superficial on the sociological component, (only represented by the question whether the participant lived alone or not, which may have many different reasons without necessarily meaning that a person is socially isolated or not), have been applied.
Unfortunately, a conclusive measure of quality of life such as HRQOL relevant for successful ageing and driving status is missing (Hajek A 2021).

Thank you for this remark. We have added “Indeed, it has even been shown that driving correlates well with health-related quality of life (HRQOL) if 544 subjects of 90.3 years (± 2.7) on self-care and usual-activities domains (OR 0.41 (95%-CI 0.17 to 0.98 OR 0.48 (0.26 to 0.90) respectively).(43)” in the discussion section (L278)

To sum up, the manuscript “Association between car driving and successful ageing. A cross sectional study on the "S.AGES" cohort” by E. Baudouin and colleagues may pose a relevant contribution to the scientific field of successful ageing. It is in line with the current literature supporting the view that driving is a relevant aspect to wellbeing until older age (in industrialised countries). The shortcomings are most likely due to the fact that the study was not primarily designed to answer the question of successful ageing but to look at GPs management of chronic conditions namely diabetes mellitus, chronic pain and atrial fibrillation. A fact that is outlined by the authors in the text and will be evaluated by the editors. If this is considered suitable, after minor revision taking into account the remarks made above a publication in PLOS One can be recommended.

We greatly thank you for your support
