# Supplementary material for: Association between car driving and successful ageing. A cross sectional study on the "S.AGES" cohort
Source: PLoS One. 2023 May 4;18(5):e0285313. doi: 10.1371/journal.pone.0285313 (PMC10159353; doi:10.1371/journal.pone.0285313)
Supplement: Attachment — Submitted filename: Re-Review PONE D-22-26799.docx [file pone.0285313.s009.docx]

Reviewer 3:

PONE-D-22-26799

“Association between car driving and successful ageing. A cross sectional study on the "S.AGES" cohort” by E. Baudouin and colleagues

The manuscript to be re-reviewed has been revised by the authors and my comments are as follows:

Thank you for considering my comments in general. Nevertheless, I want to outline some shortcomings that are still recommended to be mentioned in the limitations section. As there is the fact that only cursory and superficial aspects of cognition (only MMSE) and the sociological component (living alone or not) have been investigated to relate driving status and successful ageing, reducing the validity of the results. The authors themselves state that a more sophisticated geriatric and cognitive screening is warranted to measure cognitive domains relevant for driving, but without referring to their own study design.

In lines 225-227 the authors state that daily somnolence in the elderly is due to the use of Z-drugs (e.g. Zopiclon, Zolpidem) (ll 255-257). As, in fact, this is not the main and only cause of day time sleepiness in the elderly (neurological and somatic disordes, sleep apnea etc) this should be elucidated or omitted.

The paragraph following this statement (ll 258-264) as well as larger (esp. the revised) parts of the manuscript, are difficult to understand. Hence, proof-reading and language polishing is recommended as the readability of the original manuscript was better than of the revised version.

To sum up, the manuscript “Association between car driving and successful ageing. A cross sectional study on the "S.AGES" cohort” by E. Baudouin and colleagues may still add a relevant aspect to the scientific field of successful ageing. It is in line with the current literature supporting the view that driving is a relevant aspect to wellbeing until older age (in industrialised countries). Still, the shortcomings are most likely due to the fact that the study was not primarily designed to answer the question of successful ageing but to look at GPs management of chronic conditions. This fact will be evaluated by the editors. If this is considered suitable, after some further minor revision a publication in PLOS One can be recommended.
