# Supplementary material for: Association between car driving and successful ageing. A cross sectional study on the "S.AGES" cohort
Source: PLoS One. 2023 May 4;18(5):e0285313. doi: 10.1371/journal.pone.0285313 (PMC10159353; doi:10.1371/journal.pone.0285313)
Supplement: Attachment — Submitted filename: Response to Reviewers.docx [file pone.0285313.s010.docx]

**Editor:**

Several references have been changed since the first submission after reviews.

Please note the following additions:

- Bucher S, Al-Salameh A, Panjo H, Becquemont L, Ringa V. Looking younger, dying later: General practitioners’ intuitive clinical impression predicts mortality. Prev Med. 2018 Jun 1;111:28–34.
  - Added in order to give a better explanation and justification on the use of “physiological age”
- Blank K, Gruman C, Robison JT. Case-Finding for Depression in Elderly People: Balancing Ease of Administration With Validity in Varied Treatment Settings. J Gerontol Ser A. 2004 Apr 1;59(4):M378–84. Added
  - Provided in order to justify used for the GDS
- Rowe JW, Kahn RL. Successful Aging. The Gerontologist. 1997 Aug 1;37(4):433–40.
  - In replacement of “Rowe JW, Kahn RL. Human Aging: Usual and Successful. Science. 10 juill 1987;237(4811):143” as asked by previous review
- Bucher S, Al-Salameh A, Panjo H, Becquemont L, Ringa V. Looking younger, dying later: General practitioners’ intuitive clinical impression predicts mortality. Prev Med. 2018 Jun 1;111:28–34.
  - Added in order to justify the use and methodology of physiological age
- Crizzle AM, Mullen N, Mychael D, Meger N, Toxopeus R, Gibbons C, et al. The SIMARD-MD is not an Effective Driver Screening Tool for Determining Fitness-To-Drive. Can Geriatr J. 2021 Mar 2;24(1):14–21.
  - Added to justify the limit of current developed screening tools for driving
- Dobbs BM, Shergill SS. How effective is the Trail Making Test (Parts A and B) in identifying cognitively impaired drivers? Age Ageing. 2013 Sep;42(5):577–81.
  - Added to justify the limit of current developed screening tools for driving
- Khan MAB, Hashim MJ, King JK, Govender RD, Mustafa H, Al Kaabi J. Epidemiology of Type 2 Diabetes – Global Burden of Disease and Forecasted Trends. J Epidemiol Glob Health. 2020 Mar;10(1):107–11.
  - Added to assess diabetes prevalence
- Krijthe BP, Kunst A, Benjamin EJ, Lip GYH, Franco OH, Hofman A, et al. Projections on the number of individuals with atrial fibrillation in the European Union, from 2000 to 2060. Eur Heart J. 2013 Sep 14;34(35):2746–51.
  - Added to assess atrial fibrillation prevalence
- Cohen SP, Vase L, Hooten WM. Chronic pain: an update on burden, best practices, and new advances. The Lancet. 2021 May;397(10289):2082–97.
  - Added to assess chronic pain prevalence
- Trivedi D. Cochrane Review Summary: Mini-Mental State Examination (MMSE) for the detection of dementia in clinically unevaluated people aged 65 and over in community and primary care populations. Prim Health Care Res Dev. 2017 Nov;18(6):527–8.
  - Added to justify MMSE’s use in screening for detection of dementia
- Nosraty L, Pulkki J, Raitanen J, Enroth L, Jylhä M. Successful Aging as a Predictor of Long-Term Care Among Oldest Old: The Vitality 90+ Study. J Appl Gerontol. 2019 Apr 1;38(4):553–71.
  - Added to justify the use of social isolation in SA
- Choi M, Lee M, Lee M-J, Jung D. Physical activity, quality of life and successful ageing among community-dwelling older adults. Int Nurs Rev. 2017;64(3):396–404.
  - Added to justify the use of social isolation in SA

Please note the following removals:

- Older drivers [Internet]. IIHS-HLDI crash testing and highway safety. [cité 30 mars 2022]. Disponible sur: <https://www.iihs.org/topics/older-drivers>
  - Removed and remplaced with French statistics as asked by review
- Rowe JW, Kahn RL. Human Aging: Usual and Successful. Science. 10 juill 1987;237(4811):143‑
  - Removed and replaced by correct citation as asked by review
- Kostyniuk LP, Shope JT. Driving and alternatives: Older drivers in Michigan. J Safety Res. janv 2003;34(4):407‑14.
  - Typo during redaction, unused citation

One redundance have also been corrected:

- Rubenstein LZ, Stuck AE, Siu AL, Wieland D. Impacts of geriatric evaluation and management programs on defined outcomes: overview of the evidence. J Am Geriatr Soc. 1991 Sep;39(9 Pt 2):8S-16S; discussion 17S-18S

**Reviewer**

We thank you for your remarks and support. We addressed all the questions that you raised and highlighted it in the text in green.

Please find enclosed our answers

I want to outline some shortcomings that are still recommended to be mentioned in the limitations section. As there is the fact that only cursory and superficial aspects of cognition (only MMSE) and the sociological component (living alone or not) have been investigated to relate driving status and successful ageing, reducing the validity of the results. The authors themselves state that a more sophisticated geriatric and cognitive screening is warranted to measure cognitive domains relevant for driving, but without referring to their own study design.

We added the following in the limitation paragraph (L 276):

“Second, we acknowledge that two dimensions may be superficially explored: cognitive impairment and social dimension. Regarding the former, the sole use of MMSE might underestimate cognitive impairment, however widely used as a screening tool [50]. We also recognize that the definition used for social dimension may limit the scope of our results as it only incudes living alone or not; which may not always results in loneliness. Nonetheless, this proxy has been shown to correlated with other SA definitions [51,52].

In lines 225-227 the authors state that daily somnolence in the elderly is due to the use of Z-drugs (e.g. Zopiclon, Zolpidem) (ll 255-257). As, in fact, this is not the main and only cause of day time sleepiness in the elderly (neurological and somatic disorders, sleep apnea etc) this should be elucidated or omitted. The paragraph following this statement (ll 258-264) as well as larger (esp. the revised) parts of the manuscript, are difficult to understand. Hence, proof-reading and language polishing is recommended as the readability of the original manuscript was better than of the revised version.

This paragraph has been changed and updated in order to clarify its meaning and improve its understanding:

“The high rate of subjects who did not reached the psychological component among drivers can be noted. When suffering from dementia, mild cognitive impairment or depression, subjects may frequently have impaired attention and executive functions, or be at higher risk of using drugs that may impair driving ability [40–42]. This result may be explained by the fact that French legislation does not require regular reevaluation of driving abilities. However, we may see improvement in future cohort. Indeed, since the fall of 2022, when suffering from a specific group of diseases (including cognitive impairment), it is required for the patient to undertake a specific medical examination focused on driving abilities by a certified physician.”

The manuscript has also been proof-read by an English native speaker.
