# Supplementary material for: Association between car driving and successful ageing. A cross sectional study on the "S.AGES" cohort
Source: PLoS One. 2023 May 4;18(5):e0285313. doi: 10.1371/journal.pone.0285313 (PMC10159353; doi:10.1371/journal.pone.0285313)
Supplement: Attachment — Submitted filename: Re-Review PONE D-22-26799_4_16_2023.docx [file pone.0285313.s011.docx]

Reviewer 3:

PONE-D-22-26799

“Association between car driving and successful ageing. A cross sectional study on the "S.AGES" cohort” by E. Baudouin and colleagues

The manuscript to be re-reviewed has been revised by the authors and my comments are as follows:

Thank you for going over the manuscript again, as a result the quality of language and readability has improved substantially. Also, my content-related remarks have been considered well.

By going through it again I only came across a few spelling errors or omissions that might be corrected before publication, such as

line 263: ´reach` instead of ´reached`

line 287: `rates´ instead of `rate´

line 289: either past or present tense (see `overestimate´ and `overestimated`)

line 292: `subjects´ instead of `subject´

line 305: `impacted on´

To sum up, the manuscript “Association between car driving and successful ageing. A cross sectional study on the "S.AGES" cohort” by E. Baudouin can be recommended for publication.
