# Supplementary material for: Association between car driving and successful ageing. A cross sectional study on the "S.AGES" cohort
Source: PLoS One. 2023 May 4;18(5):e0285313. doi: 10.1371/journal.pone.0285313 (PMC10159353; doi:10.1371/journal.pone.0285313)
Supplement: Attachment — Submitted filename: Response to Reviewers.docx [file pone.0285313.s012.docx]

**Reviewer :**

Thank you for going over the manuscript again, as a result the quality of language and readability has improved substantially. Also, my content-related remarks have been considered well.

By going through it again I only came across a few spelling errors or omissions that might be corrected before publication, such as

line 263: ´reach` instead of ´reached`

line 287: `rates´ instead of `rate´

line 289: either past or present tense (see `overestimate´ and `overestimated`)

line 292: `subjects´ instead of `subject´

line 305: `impacted on´

To sum up, the manuscript “Association between car driving and successful ageing. A cross sectional study on the "S.AGES" cohort” by E. Baudouin can be recommended for publication.

We thank you for your close reading and support. Corrections have been made as suggested and are highlighted in blue.

**Editor:**

Several references have been changed since the first submission after reviews.

Please note the following additions:

- Bucher S, Al-Salameh A, Panjo H, Becquemont L, Ringa V. Looking younger, dying later: General practitioners’ intuitive clinical impression predicts mortality. Prev Med. 2018 Jun 1;111:28–34.
  - Added in order to give a better explanation and justification on the use of “physiological age”
- Blank K, Gruman C, Robison JT. Case-Finding for Depression in Elderly People: Balancing Ease of Administration With Validity in Varied Treatment Settings. J Gerontol Ser A. 2004 Apr 1;59(4):M378–84. Added
  - Provided in order to justify used for the GDS
- Rowe JW, Kahn RL. Successful Aging. The Gerontologist. 1997 Aug 1;37(4):433–40.
  - In replacement of “Rowe JW, Kahn RL. Human Aging: Usual and Successful. Science. 10 juill 1987;237(4811):143” as asked by previous review
- Bucher S, Al-Salameh A, Panjo H, Becquemont L, Ringa V. Looking younger, dying later: General practitioners’ intuitive clinical impression predicts mortality. Prev Med. 2018 Jun 1;111:28–34.
  - Added in order to justify the use and methodology of physiological age
- Crizzle AM, Mullen N, Mychael D, Meger N, Toxopeus R, Gibbons C, et al. The SIMARD-MD is not an Effective Driver Screening Tool for Determining Fitness-To-Drive. Can Geriatr J. 2021 Mar 2;24(1):14–21.
  - Added to justify the limit of current developed screening tools for driving
- Dobbs BM, Shergill SS. How effective is the Trail Making Test (Parts A and B) in identifying cognitively impaired drivers? Age Ageing. 2013 Sep;42(5):577–81.
  - Added to justify the limit of current developed screening tools for driving
- Khan MAB, Hashim MJ, King JK, Govender RD, Mustafa H, Al Kaabi J. Epidemiology of Type 2 Diabetes – Global Burden of Disease and Forecasted Trends. J Epidemiol Glob Health. 2020 Mar;10(1):107–11.
  - Added to assess diabetes prevalence
- Krijthe BP, Kunst A, Benjamin EJ, Lip GYH, Franco OH, Hofman A, et al. Projections on the number of individuals with atrial fibrillation in the European Union, from 2000 to 2060. Eur Heart J. 2013 Sep 14;34(35):2746–51.
  - Added to assess atrial fibrillation prevalence
- Cohen SP, Vase L, Hooten WM. Chronic pain: an update on burden, best practices, and new advances. The Lancet. 2021 May;397(10289):2082–97.
  - Added to assess chronic pain prevalence
- Trivedi D. Cochrane Review Summary: Mini-Mental State Examination (MMSE) for the detection of dementia in clinically unevaluated people aged 65 and over in community and primary care populations. Prim Health Care Res Dev. 2017 Nov;18(6):527–8.
  - Added to justify MMSE’s use in screening for detection of dementia
- Nosraty L, Pulkki J, Raitanen J, Enroth L, Jylhä M. Successful Aging as a Predictor of Long-Term Care Among Oldest Old: The Vitality 90+ Study. J Appl Gerontol. 2019 Apr 1;38(4):553–71.
  - Added to justify the use of social isolation in SA
- Choi M, Lee M, Lee M-J, Jung D. Physical activity, quality of life and successful ageing among community-dwelling older adults. Int Nurs Rev. 2017;64(3):396–404.
  - Added to justify the use of social isolation in SA

Please note the following removals:

- Older drivers [Internet]. IIHS-HLDI crash testing and highway safety. [cité 30 mars 2022]. Disponible sur: <https://www.iihs.org/topics/older-drivers>
  - Removed and remplaced with French statistics as asked by review
- Rowe JW, Kahn RL. Human Aging: Usual and Successful. Science. 10 juill 1987;237(4811):143‑
  - Removed and replaced by correct citation as asked by review
- Kostyniuk LP, Shope JT. Driving and alternatives: Older drivers in Michigan. J Safety Res. janv 2003;34(4):407‑14.
  - Typo during redaction, unused citation

One redundance have also been corrected:

- Rubenstein LZ, Stuck AE, Siu AL, Wieland D. Impacts of geriatric evaluation and management programs on defined outcomes: overview of the evidence. J Am Geriatr Soc. 1991 Sep;39(9 Pt 2):8S-16S; discussion 17S-18S
